# 892. Determination of the Unavailability of Alternative Antiretroviral Formulations

**DOI:** 10.1093/ofid/ofab466.1087

**Published:** 2021-12-04

**Authors:** Milena M Murray, Devon Flynn, Leonard A Sowah, Aaron Austin, Eric Farmer

**Affiliations:** 1 Midwestern University - Chicago College of Pharmacy, Downers Grove, Illinois; 2 Oregon Health & Science University, Portland, Oregon; 3 National Institute of Allergy and Infectious Diseases, Rockville, Maryland; 4 American Academy of HIV Medicine, Pensacola, Florida; 5 Indiana University Health LifeCare Clinic, Indianapolis, Indiana

## Abstract

**Background:**

Many pediatric and some adult people living with HIV (PLWH) are unable to swallow tablets and require alternative antiretroviral formulations (ARVF) such as liquids, chewable tablets, or powders for suspension. A growing number of issues with the timely procurement of alternative ARVF have been reported; the full scope of this problem is unknown. Without access to appropriate treatment, PLWH are at increased risk of poor disease outcomes. This study’s objective was to determine the scope of availability issues of ARVF and its potential impact on patient care.

**Methods:**

An online survey invitation was sent to members of AAHIVM and the ACCP HIV PRN. Data collection included provider demographics, number of issues related to ARVF availability, time spent procuring ARVFs, and identification of unavailable formulations. To determine potential impact on clinical care and cost of care the time required to resolve shortages was summarized.

**Results:**

The analyzable sample was 154, a majority of whom were pharmacists or physicians (n=132, 85.7%; Figure 1), in a clinical role (n=134, 87.0%), and serve pregnant patients (n=121, 79.2%). 85 (55.2%) practice at sites that provide care to > 300 PLWH, 81 (52.6%) practice at sites that did not serve pediatric patients. 525 instances of gaps in care due to ARVF unavailability were reported. In 283 instances, a more complex regimen was prescribed due to first-choice ARVF unavailability. Providers also reported 186 situations in which a less optimal regimen was used and 140 cases of treatment delays. The average time spent to resolve such issues was 2.7 hrs (CI: 1.3 – 4.2). The longest time reported was 72 hrs; most providers spent 1 hr or less. The most common unavailable ARVF were branded ritonavir 80 mg/mL solution (n=12), zidovudine 50 mg/5 mL syrup (n=11), raltegravir 100 mg chewable tablets (n=11), and raltegravir 100 mg granules for suspension (n=10). Branded nevirapine 50 mg/5 mL suspension (n=7) and generic nevirapine 50mg/5ml powder for suspension (n=11) were also reported more frequently.

Distribution of Respondents by Provider Type

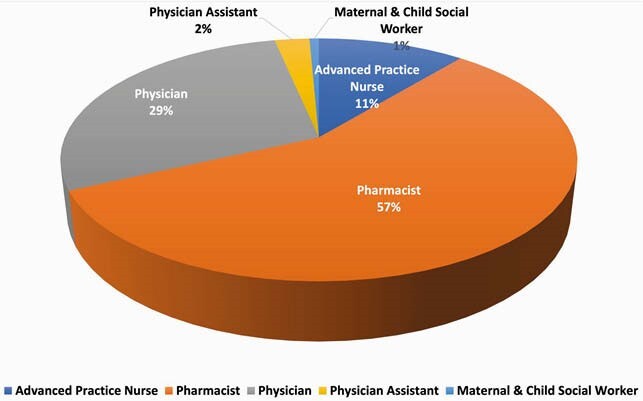

**Conclusion:**

Our report suggests the unavailability of alternative ARVF has the potential to significantly impact patient care. Further research is needed to identify the root causes of this problem to determine specific solutions.

**Disclosures:**

**Milena M. Murray, PharmD, MSc, BCIDP, AAHIVP**, **Merck** (Speaker’s Bureau)**Theratechnologies** (Other Financial or Material Support, Medical Advisory Board) **Eric Farmer, PharmD, BCPS, AAHIVP**, **TheraTechnologies, Inc** (Other Financial or Material Support, Medical Advisory Board)

